# Health and healthcare access among Zambia’s female prisoners: a health systems analysis

**DOI:** 10.1186/s12939-016-0449-y

**Published:** 2016-09-26

**Authors:** Stephanie M. Topp, Clement N. Moonga, Constance Mudenda, Nkandu Luo, Michael Kaingu, Chisela Chileshe, George Magwende, Jody S. Heymann, German Henostroza

**Affiliations:** 1 College of Public Health Medical and Veterinary Sciences, James Cook University, Townsville, 4812 Australia; 2 Centre for Infectious Disease Research in Zambia, PO Box 30346, Lusaka, Zambia; 3 c/- CAPAH, National Assembly Parliament Buildings, PO Box 31299, Lusaka, Zambia; 4 ZPS Headquarters, PO Box 80926, Kabwe, Zambia; 5 School of Public Health, University of California, LA, Los Angeles, CA USA; 6 University of Alabama at Birmingham, Birmingham, AL USA

**Keywords:** Women prisoners, Prisoner health, Prison health services, Health systems

## Abstract

**Background:**

Research exploring the drivers of health outcomes of women who are in prison in low- and middle-income settings is largely absent. This study aimed to identify and examine the interaction between structural, organisational and relational factors influencing Zambian women prisoners’ health and healthcare access.

**Methods:**

We conducted in-depth interviews of 23 female prisoners across four prisons, as well as 21 prison officers and health care workers. The prisoners were selected in a multi-stage sampling design with a purposive selection of prisons followed by a random sampling of cells and of female inmates within cells. Largely inductive thematic analysis was guided by the concepts of dynamic interaction and emergent behaviour, drawn from the theory of complex adaptive systems.

**Results:**

We identified compounding and generally negative effects on health and access to healthcare from three factors: i) systemic health resource shortfalls, ii) an implicit prioritization of male prisoners’ health needs, and iii) chronic and unchecked patterns of both officer- and inmate-led victimisation. Specifically, women’s access to health services was shaped by the interactions between lack of in-house clinics, privileged male prisoner access to limited transport options, and weak responsiveness by female officers to prisoner requests for healthcare. Further intensifying these interactions were prisoners’ differential wealth and access to family support, and appointments of senior ‘special stage’ prisoners which enabled chronic victimisation of less wealthy or less powerful individuals.

**Conclusions:**

This systems-oriented analysis revealed how Zambian women’s prisoners’ health and access to healthcare is influenced by weak resourcing for prisoner health, administrative biases, and a prevailing organisational and inmate culture. Findings highlight the urgent need for investment in structural improvements in health service availability but also interventions to reform the organisational culture which shapes officers’ understanding and responsiveness to women prisoners’ health needs.

## Background

In sub-Sahara, African women prisoners constitute between 1 and 4 % of the total prison population [1]. Although evidence remains thin in low-income settings, peer reviewed literature from high-income settings demonstrates that women prisoners experience higher rates of emotional, physical and sexual abuse compared with non-incarcerated women [2]. Evidence from a number of (predominantly high-income) countries also highlights the fact that women prisoners tend to experience higher rates of physical and mental disease while incarcerated compared to their male counterparts [3–6]. Marginalisation is due to a range of structural, relational and demographic factors compounded by weak advocacy for, or inclusion of women prisoners’ needs in domestic public policy debates [7, 8]. Despite their small absolute numbers, women prisoners represent the fastest growing incarcerated population globally and have seen a 22 % increase in sub-Saharan African prisons since 2000 [9, 10]

In sub-Saharan Africa, prisons research as a whole is lacking, and empirical research focussing on the experiences and issues of women prisoners is almost non-existent [7]. A few notable exceptions include a study from four Zambian prisons, which found women prisoners were underserved by general healthcare programs and heavily impacted by physical and sexual abuse [11]. In a mixed methods study in one Ghanaian prison, Sarpong *et al* [12] reported women prisoners had poor access to quality healthcare, noting demographic characteristics, marital status, educational background and occupation influenced respondents’ perceptions of, and access to services. In South Africa several studies have documented aspects of women prisoners’ experience including the carceral space, the role of trauma [5] and the implementation of health policies [13]. Although growing concerns about HIV and TB epidemics in prison populations have also resulted in several recent studies demonstrating high rates of infectious diseases in sub-Saharan African prisons [14–17], none of these report fully gender-disaggregated data making them less useful for understanding women prisoners’ disease burden or healthcare needs [18].

The ability of policy makers and programmers to develop sophisticated and sustainable interventions to address the complex needs of female prisoners remains constrained by lack of empiric data documenting epidemiological patterns and the institutional and social dynamics influencing female prisoners’ health and access to health care. While it is generally understood that environmental conditions are dire, for example, and that women’s psychological and physical health needs are likely to be being overlooked, evidence of how structural conditions interact with social and institutional factors in the facility to influence women prisoners’ behaviours or health service access in these settings is largely lacking. This study was designed to expand the evidence base in Zambia regarding women prisoners’ health and access to healthcare.

## Methods

### Study setting

Zambia has 87 prisons in total of which 54 are considered ‘conventional’ holding facilities and the remainder termed ‘farm’ or open-air prisons. Of the 54 conventional facilities, one is female only, one is a juvenile facility, and the remainder have both a male and female wing. Of these 52 conventional facilities, 45 have a ‘built-for-purpose’ female wing. For the remaining seven facilities, female accommodation is makeshift. Regardless of facility type, female prisoners in Zambia have been exclusively under female guard since 2001. At the time of data collection (August – November 2014), the female population was approximately 480 (Personal Communication), 2.7 % of the total Zambian prison population. Site-specific populations ranged from as few as 4–5 to as high as 250 women prisoners.

Zambian prisons are administrated by the Zambian Prison Service (ZPS) under the Ministry of Home Affairs. Evidence from several recent epidemiological studies, demonstrate that both male and female prisoners in Zambia experience high rates of communicable disease [14, 19]. In 2011, a cross-sectional study assessing rates of TB in six prison facilities (three holding women inmates) and adjacent residence (mainly comprising prison personnel and their families) found a rate of confirmed TB amongst females of 2.7 % (comprising 1.2 % bacteriologically confirmed and 1.5 % clinically diagnosed) [20].

In 2013, ZPS, the Ministry of Health (MOH), Ministry of Community Development Mother and Child Health (MCDMCH) with support from the Centre for Infectious Disease Research (CIDRZ) and the United National Office for Drugs and Crime (UNODC) embarked on an ambitious three-year program to strengthen the prison health system [21–23]. To help inform this process and provide stakeholders with current evidence, a research component was built into the project to better understand the health and health care experiences of both male and female prisoners. This study reports findings from the female-focused component of the larger research project. Previous work has reported on other aspects of this research including structural barriers to Zambian prison health system improvements [24] and the experiences of Zambian male prisoners [25]. Finally we note that for the purpose of this paper we use the terms ‘inmate’ and ‘prisoner’ interchangeably; where relevant we distinguish between those who are ‘on remand’ and those ‘convicted’.

This study aimed to explore and describe the interactions between structural and relational factors influencing female prisoners’ health, health risks, and access to health care, with the overall goal of improving prison health in Zambia. The study was cross-sectional in design and guided by an understanding of health systems – including prison health systems – as not simply mechanistic delivery systems, but social systems characterized by multiple actors [26] and localized social and political power structures [27, 28]. Our selection of qualitative methods was designed to enable an exploration of these (as yet imperfectly understood) features in the Zambian setting, and their interactions with other, broader contextual factors (e.g. structural, material and relational). Our overall aim was to develop a deeper understanding of the factors underpinning female prisoners’ health and health service access.

### Study population and sampling

We conducted in-depth interviews of a clustered random sample of female prisoners in a purposeful sample of prisons, as well as prison guards and health care workers in the same sites. A total of 44 interviews were conducted, including 23 women prisoners and 21 officers and healthcare workers. Four Zambian prisons were purposively selected based on geographic spread (one facility in each of four provinces), and a range of security levels (two medium security, one maximum security and one low-security District facility). Each site had a recruitment target of 5–6 female prisoners (including three known to be HIV positive) and five prison officers. Recruitment targets resulted in a minimum of 6 % (Facility 2) and a maximum of 25 % (Facility 4) of the total female population at each site being sampled as well as ensuring sufficient data on experiences from very different facility types. Participant inclusion criteria included having lived or worked in the selected prison site for three months or more, and being capable and willing to provide informed verbal consent. We excluded those under 18 years of age (Zambia’s legal age of consent) and those with a known history of mental illness.

Sampling of prisoners at the facility level was carried out using a multi-stage sampling design. The principal investigator (PI) first identified the total number of cells (typically between 4 and 6) and then randomly selected two. From a list of prisoners per cell (typically between 4 and 6), the PI selected three individuals by first randomly selecting a ‘starting’ number from a hat, and then sampling every fifth female on the list from that number. Using the same approach (i.e. random selection of a starting number followed by selection of every fifth listed individual), three additional prisoners were selected from a full list of prisoners recorded as receiving HIV care and treatment. Where an inmate was unavailable due to illness, assigned labour duties or other reasons, the next (fifth) individual in sequence was selected from the appropriate list. Recruitment of prison officers was purposive (based on rostered staff lists) and designed to ensure a mix of interviews with senior management, non- ranking prison officers and professional health personnel working at the prison clinic or nearby public health centre.

### Recruitment and interview procedures

Data collection was carried out by a pair of multi-lingual Zambian research assistants (RAs), working as a team. The RAs were recruited based on previous experience conducting sensitive in-depth interviews and received an intensive five-day training encompassing human subjects protection, familiarisation with the study’s aim and the study tools, the Zambian prison context, and best-practice approaches to qualitative interviewing.

With prior permission from the Commissioner of Prisons and the Officer in Charge the PI worked closely with the prison nurse or clinical officer to make arrangements for the removal of randomly selected inmates to a nominated venue within each prison. Security protocol meant that inmates could only be identified and accompanied from their cells by an officer. In all four facilities, interviews were conducted in closed-door rooms.

To ensure participant protection we adopted a verbal consent protocol. Special care was taken to both offer voluntary participation and, to minimise staff or other inmates’ knowledge of any individual’s participation in the study. Potential participants were provided clear information that the study was not linked to medical treatment or any other service. Verbal consent was witnessed by an independent lay health worker recruited from the closest public health centre. All participants were offered a copy of the study information sheet but were not obliged to take or keep a copy if they felt it would compromise their confidentiality. Interview participants were not paid or incentivised to participate. Ethical clearance was gained from the University of Zambia Biomedical Research Ethics Committee and the University of Alabama at Birmingham IRB.

Interviews were carried out in the participant’s choice of English or one of five local languages (Nyanja, Bemba, Tonga, Lozi, Kunda) and were between 30 min and one hour in length. Interview guides included questions on topics that investigator experience and the broader literature on prisoner experience have shown to be important to female prisoners’ health. Questions covered both factual and chronological detail about day-to-day life as well as perceptions and experiences and social relations.

### Data management and analysis

Analysis began during field work with reflective, investigator-led debriefing sessions at the end of each day of interviewing. Important and emergent themes or topics were noted and incorporated into subsequent interviews and summary notes transcribed and incorporated into in-depth analysis. A final debriefing workshop to discuss cross-facility similarities and differences was conducted after the completion of all fieldwork. All interviews were audio-recorded, and later transcribed and translated into English (where necessary) in a single step. Transcripts were imported into NVivo QSR™ and read twice in full prior to a draft code-book being developed. Two rounds of coding were conducted, with codes refined during the process. Draft findings and interpretations were reviewed and member-checked by two other investigators at each stage of an iterative process. Codes were gradually consolidated and grouped into larger themes relating to inmate health and healthcare. A summary of draft findings was additionally circulated within the Zambian Prison Service to garner feedback. Our approach was guided by recognized qualitative analysis techniques including reading for content, coding, data reduction, data display, and interpretation [29, 30].

## Results

A total of 44 interviews were conducted comprising 23 female inmates and 21 prison officials. Table 1 below describes basic demographic characteristics for the inmates and the breakdown of interviews conducted in each of the four study sites.

**Table 1:** Demographic characteristics of interview respondents in four prison facilities^a^

Inmates	Facility 1	Facility 2	Facility 3	Facility 4	Total
Female	5	6	6	6	23 (100 %)
Convicted (%)	4 (80 %)	5 (83 %)	5 (83 %)	3 (50 %)	17 (74 %)
Mean Time Served (Months)	13	63	42	5	33
Mean Age (Years)	29	35	28	36	33
Ever Married (%)	3 (60 %)	5 (83 %)	5 (83 %)	6 (100 %)	19 (83 %)
Looking after child in prison	3 (60 %)	0	2 (33 %)	2 (33 %)	7 (30 %)
HIV-positive	3 (60 %)	3 (50 %)	3 (50 %)	2 (33 %)	18 (78 %)
Officers / Other Staff	Facility 1	Facility 2	Facility 3	Facility 4	
Female officers	3	2	5	6	16
Healthcare workers	1	1	2	1	5
Total Interviews /Facility	4	3	7	7	44

^a^Total female inmate population in Zambia at time of study ~238

All 23 female prisoners (100 %) reported feeling anxious about health or access to healthcare in prison, with five major themes emerging. These were: i) environmental conditions; ii) nutrition and cooking arrangements; iii) social networks and relationships; iv) healthcare access and v) responsibility for children. Findings from these five overarching themes will be detailed in sequence.

### Environmental conditions

In all sites, female inmates reported poor environmental conditions with particular emphasis on poor sleeping conditions and terrible sanitation.

#### Overcrowding and sleeping conditions

In one of the four sites, female cells were converted store houses adjacent to the original, all-male prison. In three sites, female cells were purpose built. Overcrowding was most pronounced in Facilities 1 and 2 (the sites with the largest female populations) with cells designed for four people typically housing between 8 and 10 women. In these same sites, inmates slept on thin mats on the floor. Ventilation was poor with small grids high in the walls and a number of inmates described their anxiety about the risk of airborne infectious diseases, particularly tuberculosis (TB).

*The [cells] are very bad, especially sleeping, it is very bad. In one cell, there are a lot people and there isn’t enough air to breathe. Windows are too small, you can’t get enough air inside. [Female, Facility 1]*

*You know the cells are currently very full. So if I have TB, coughing all night, and am in the same room with those that don’t have it, they will eventually catch it [Female, Facility 2]*

Overcrowding was reported to be less of a problem in Facilities 3 and 4. Although inmates reported sleeping two to a mattress, for example, there was sufficient space to hang mosquito nets.

#### Sanitation and hygiene

Insufficient toilets, broken toilets or lack of access to toilets (at night) were mentioned by 20 of 23 inmate respondents as negatively affecting their health. Inability to keep toilets clean, through overuse and lack of cleaning products were mentioned by respondents from all sites, but seemed particularly acute in Facilities 1 and 2.

*The bathroom is okay but not the toilet because there’s just one and there are 20, sometimes 25 of us in the cell. [Female, Facility 3]*

*Yes I had sores in between my buttocks because of sharing toilets and in a crowd like this. So I didn’t know that I could catch a disease through sitting on the toilet seat. I would wipe it with toilet paper then sit on it, I didn’t know that my fellow inmates were not sitting but squatting on it. [Female, Facility 2]*

Almost all the women interviewed described some anxiety regarding the potential for infectious disease transmission resulting from inadequate sanitation, particularly skin infections, dysentery, and lice. Soap, detergent and sanitary products were not supplied by the prison.

Sanitary products were obtained from various sources. Frequent, but *ad hoc* donations of disposable sanitary pads were made by various Church groups at all sites, and some women also reported receiving gifts of disposable pads from visiting friends or family. In two sites (2, 3), however, several respondents reported never having received or having run out of disposable pads forcing them to rely on reusable cloth pads hand-made by themselves from cotton wool and scrap cloth donated by the Church. Contributing to this shortage, in two sites (1, 2) women reported some officers forcing them to hand over a proportion of any gifts of pads on multiple occasions; members of the study team observed this occurring at one site. Disposable pads were thrown out in the daily garbage (with no special infection control measures) while re-usable pads had to be washed and dried daily despite the scarcity of laundry soap. In one site (1) where fairly regular Church donations of second hand clothing made it possible, women made reusable pads for sale or barter with members of the local community for items including cooking oil and body lotion.

### Nutrition and cooking arrangements

All 23 women in this study mentioned insufficiency of prison rations or poor quality of prison food as a health concern.

*I think with the kind of food that we eat I can develop illnesses. The fish we eat has a lot of stones and the mealie-meal we use is not well taken care of. [Female, Facility 4]*

*There is no food here. There is no proper food. We only eat the stony kapenta every day. Even rice, you can see here where we are, there are many of us, yet there is only a small pot. [Female, Facility 2]*

*The diet is not good and if you don’t have relatives who visit, then you only be eating kapenta and beans. [Female, Facility 3]*

Meals were typically as follows. Breakfast constituted unsalted porridge or ‘samp’ served around 08:00. Lunch, constituting nshima (cooked corn meal) and beans and/or small dried fresh-water fish (kapenta) served between 12:00 and 16:00 depending on the facility. In Facilities 1 and 2, supper was an extra portion served at the same time as lunch and saved for later. In Facility 3, food was required to be eaten outside the cell resulting in lunch and supper typically being a merged meal. In Facility 4, inmates reported no extra serving for supper and additionally noted that broken cooking facilities meant that at the time of study they were also not receiving breakfast porridge. In Facility 3 and 4, portions of kapenta were handed to inmates uncooked, requiring them to either independently source firewood and/or charcoal and cooking oil or otherwise eat the dried fish raw. Constituting a significant food sanitation risk, inmates in three sites reported ‘saving’ their last meal of the day (served prior to the afternoon lock-up) by wrapping in re-used plastic bags and then placing it under blankets to keep it warm.

The only protein in the women’s rations was kapenta, along with occasional church donations that included soya chunks. Women frequently mentioned diarrhoea, weight loss and lethargy related to poor diet. Poor nutritional status due to lack of vegetables was also reported by a number of respondents – but was described in particularly acute terms by women with chronic illness.

*We are advised at the clinic that we should be eating vegetables. But if you don’t have visitors then [you get] no vegetables. So we can’t afford to eat what we are told at the clinic. [Female, Facility 1]*

Several respondents reported begging or trading food. However, the relatively small numbers of women within each prison meant that opportunities for internal trade were limited. Some described using cash in their ‘docket’ (personal belongings kept in trust by prison officers) to purchase sugar and cooking oil, but noted that this was dependent on the good will of an officer and often resulted in the loss of any change.

#### Cooking equipment

In all four prisons, women were responsible for cooking at least their own lunch and suppers and sometimes breakfast as well. Respondents described a limited number of stoves and cooking implements (pots, pans, spoons) which made preparing prison rations or externally sourced food-stuffs difficult. All equipment was stored outside the sleeping cells. Since women often had compulsory chores and scheduled times for bathing, time for cooking was limited resulting in frequent bottlenecks to access equipment. If cooking was not completed before lock-up inmates were not able to eat.

*We only [have] two stoves, and four of us have to use one [electric] plate. One person will want to prepare lunch, supper and breakfast and there’s only one [electric] plate. So sometimes we go inside without eating. [Female, Facility 1]*

### Social networks, relationships

#### Inmate hierarchy

Inmate health and health seeking behaviours were influenced by a range of social and relational factors. Primary among these was an inmate ranking system, which afforded some female inmates significant privileges and power over others – as outlined in Table 2.

**Table 2:** Female inmate hierarchy

Inmate hierarchy in both Zambian male and female prisons is supposed to be based on a combination of time served and good behaviour that enables ‘promotions’ through various ‘stages’ (1-5) of the hierarchy. The highest, Stage 5 or ‘Special Stage’ appointments are made on the recommendation of an Officer in Charge and are ratified by the Prisons Commissioner. Notably, all Special Stage appointments come with responsibilities and privileges including access to the officers, substantially better sleeping arrangements and the ability to deputise other inmates to carry out certain duties. Special Stagers are responsible for deputising ‘Cell Captains’ who maintain discipline within individual cell blocks and who have the authority to report ‘cases’ of indiscipline. Cell Captains are also responsible for the management of illness within their cell including identification of sick inmates and facilitating referrals to the clinics. Cell Captains typically have sleeping privileges and greater freedom of movement including easier access to internal health services (where they existed). However, these are by arrangement with the Special Stage rather than formalised rights.

**Table 3:** Barriers to accessing healthcare: experience of a HIV-positive inmate

**I:** If you need medical care, what is the process you go through to get access to it?
**P:** We ask the prison officers to take us to the hospital.
**I:** So how does it start?
**P:** We raise our hands.
**I:** What time is that done?
**P:** In the morning. In my case, I tell them that officer, my medicine has run out so I need to go to the hospital for more. They agree, the next morning we even leave the [female] prison premises, but when we get to the Male Medium prisons [to access the car], we are told there is no transport. Then we return and come and sleep without taking medicine.
**I:** Does that happen a lot?
**P:** Yes.
**I:** How long have you stayed without taking medicine?
**P:** I have gone for as long as 3 days without taking medication.

Inmates consistently described Special Stage and Cell Captains as the most influential. This influence included the authority to physically discipline or punish other inmates by leave of the officers.

*The influential ones are the captains. Those are the people that don’t treat others well. Our captain, she likes punishing people. She likes scolding people, even in her cell. She is a leader, she is a captain, [and makes] people sleep on the floor the whole night. You know what it is like to sleep on the floor when it is cold? She would punish people unnecessarily, she would do all kinds of things. [Female, Facility 1]*

*If it is announced that [members of] our cell have to go out to sweep, I sometimes would go to a prison officer to tell her that I am not well […]. But the Special Stage will be shouting at me, saying: “Let’s go outside, you malingerer! Am I the one that brought you here? Was I there when you were selling drugs? You and your illness, you just go outside to sweep!” [HIV+ Female, Facility 2]*

A number of respondents in Facilities 1, 2 and 4 described how officers and senior inmates colluded to ensure they accessed the best items when Churches or NGOs made donations.

*People from church will bring things to help us. But you find that the [officers] get some of the things and take to their homes, then they give what’s left to the Captains who get those things they want. Even baby clothes, or older people’s clothes are brought in and the [officers] and captains chose the best and leave us the useless ones. There’s always a shortage and we start fighting over the same. [Female, Facility 1].*

The power dynamics embedded in the inmate hierarchy had both direct and indirect implications for female inmates’ health and health seeking behaviours. A number of inmates described how long-serving and senior inmates had privileged access to services due to their closer relationship with officers.

*No we are not all treated the same. Some of [the officers] have favourites. [Female, Facility 2]*

*Others are scared of speaking [to officers] because they are new. For those of us that came a long time ago, we are free tell the officers when we need to seek medical care. But others, when they report that they are unwell, they are told [by the officers] that they will go to the clinic the next day. But then the next day the illness may have progressed you see, and sometimes they are not taken to the clinic, even then. [Female, Facility 2.]*

In Facilities 2 and 3, several inmates reported being forced to continue work duties despite reporting feeling ill. These commands were enforced by officers and at other times by senior inmates.

*That is the order of the day. Even when one is unwell, you will still be meant to work. [Female, Facility 2]*

*When you are not feeling well, even if you tell them you can’t work, they will still force you to go and water the gardens. [Female, Facility 3]*

#### Cooking groups & prison activities

In all four sites, women typically reported belonging to a ‘group’. Groups were described as the social locus of inmates’ lives both by choice and necessity as they tended to determine access to limited food stuffs and cooking equipment. In two sites (Facility 1 and 4) groups were formed compulsorily by female officers, with each new inmate assigned to a group. In these facilities, groups included inmates from a mix of backgrounds, and consequently, varied access to external resources. In Facilities 2 and 3, however, groups were formed voluntarily and were more selective (and inequitable) by nature. Respondents from both Facility 2 and 3, for example, described how belonging to a group became difficult if one did not have food or other material goods to share and/or exchange.

*When a new person comes, the group can agree that you will be in a group. But some are choosy, if they realise that, for instance, I don’t have people that visit me from outside, then no one will agree to be in the same group with me. If you find somebody to be in the same group with you who doesn’t have visitors, probably just for two to three days, then that will be it. They will start discouraging the new inmate from being in the group. That inmate is poor. No one visits her. She will be discouraged from being there. [Female, Facility 2]*

In two facilities, women received assistance from Church groups to participate in income-generating activities such as crocheting bath-mats, to produce saleable goods. Three facilities (2, 3, 4) had a women’s netball team that played a weekly match against prison guards. Unlike the male facilities, however, no formal educational or skills-building opportunities were provided for women prisoners at any of the sites.

#### Visitors & social isolation

Visits from family or friends were emphasised by almost all our respondents as a critical source of social and material support. Despite this, a majority of the women interviewed in this study (*n* = 15/23) reported never or rarely receiving visitors.

*My family doesn’t visit. l have no one to help me buy medicine, so we just get well by the grace of God. [Female, Facility 2]*

*Some of us don’t have relatives to visit us. They stay very far. So things we need like soap and vegetables, we don’t have. [Female, Facility 3]*

*My relatives don’t usually come because it’s very far and there is no transport. [Female, Facility 4]*

*P: No, no one has ever come.*

*I: All the 3 years you have stayed here?*

*P: No one. [Female, Facility 1]*

Descriptions of lack of visitors were imbued with a strong sense of social isolation and abandonment. Women frequently described their separation from family and the lack of visitors as traumatic, and frequently referred to concerns for children left at home. Many women reported the long distances and the time and cost associated with visiting as prohibitive. A number additionally explained how their imprisonment and/or transfer to prisons far from their families acted as a fundamental barrier to receiving visitors and the implied material support that came with them. As outlined below, however, requests for transfers back to towns-of-origin appeared to be rarely considered.

*Even when one asks for a transfer, to serve the sentence in your home town, you would be punished. There were two inmates that asked for transfers, they were punished. They don’t allow transfers here. So that makes us live in fear. [Female, Facility 3.]*

### Prison health services

#### Healthcare access

Female inmates’ access to health services was variable. Several respondents described their access to healthcare in prison as better than their experiences outside prison. These women emphasised the prioritised care received by prisoners at public clinics – not having to wait in queues – and the guarantee of free services.

*Sometimes you go to the clinic in the morning and get back home after 14:00. But in prison, immediately we go to the clinic, we are taken as the first priority. [Female, Facility 1].*

*The current treatments are better […] I am saying so because, we don’t pay, we are attended to for free. They do dental cleaning without charging us. [Female, Facility 2]*

Notwithstanding these positive accounts, 19 of the women interviewed stated that health service access was worse inside compared to outside prison. A key structural factor was the lack of internal health facilities; no female prison in this study – or indeed in Zambia – has an internal clinic. Limited availability of officers to accompany women prisoners to external health services was thus the most frequently cited problem in relation to accessing healthcare.

*We raise our hands during parade, but sometimes even when we raise our hands, we are told that there is no personnel. Then you just sit back while you are unwell. [Female, Facility 4]*

For most women, access to hospitals, which were typically further away than primary health centres, was particularly problematic since there was a need for transport and fuel as well as an accompanying officer. In several cases, women reported being told by officers that they would not be taken to the hospital unless they themselves were able to raise the cost the petrol. Several officers acknowledged during interviews they were more likely to make hospital trips when there was more than one person in need, thereby justifying the use of the vehicle and expense of the fuel.

Access to services was particularly limited in Facility 2 due to being part of a prison complex incorporating three other (all-male) prisons. Here, health resources were pooled across the four prisons that formed the larger complex, resulting in the health needs of the (numerically few) female prisoners being unintentionally but systematically deprioritised. For example, female inmates’ access to the prison clinic was just two days a week, one of which included Sundays when health providers worked truncated hours or occasionally not at all. Remaining days of the week were allocated exclusively to male inmates as described by one respondent below.

*I: What happens if you fall ill and need to go to the clinic?*

*P: Aaaaah. Then you will be told that is not the day for clinic trips. Or if it is your day and are taken there but then you find male inmates there, then you will be returned, saying there are male inmates there being treated [who have] priority.*

*I: So, it doesn’t matter the severity of the illness?*

*P: No, it doesn’t, you will be returned to the prisons.*

*I: Do male inmates have different days they go to the clinic from you female inmates?*

*P: Yes.*

*I: So, when are the womens’ days?*

*P: Fridays and Sundays.*

*I: And the rest of the days are for male inmates?*

*P: Yes.[Female, Facility 2]*

In Facility 2, female inmates’ access to external services was also limited, being reliant on a single vehicle within the complex allocated for health care referrals and court bookings. This vehicle was located at, and primarily used by officers serving the male prisons. Without a booking system, officers in the male facilities had automatic priority over its use. Officers working in the female prisons consistently reported the difficulties in sourcing transport for female inmates in order to fulfil both routine and emergency referrals.

*There are no resources, when you have to take prisoners to the hospital, […] then you are wondering how you are going to help these people? So that makes me dread work […] even fuel you have to go and beg from other services.[Female Officer, Facility 2]*

Indeed one female officer in facility 2 described taking personal initiative in flagging down members of the public and paying them out of pocket – effectively hitchhiking – or co-opting individuals or businesses hiring prisoners for farm labour in order to help transport female prisoners to the hospital.

*For me l think the most hectic one is when we are taking prisoners to the clinic. We don’t have transport. So we do panic, us officers. You just have to look for transport yourself as an officer taking those prisoners to the hospital. You have to panic […] Sometimes we go to look for free [civilian] transport; and there are people who come to hire prisoners for work so we do ask those people to assist us sometimes with transport. [Female Officer, Facility 2].*

### Officer attitudes

Among the 16 female officers interviewed, most demonstrated a good understanding of female inmates’ rights to healthcare. Notwithstanding officers’ assurances that women had the right to access healthcare any time they needed it, and as reflected in the sections above, both inmate and officer interviews revealed a series of structural barriers to meeting those rights including lack of available health services, poor transport access and insufficient officer numbers to enable (required) accompaniment.

In addition to these resourcing barriers, however, prisoner accounts also pointed to some officers’ attitudes as a substantial barrier to accessing services. Inmate accounts consistently highlighted variability in individual officers’ attitudes including accounts of those who were both supportive and those who were less so. Full-time health care workers were more frequently acknowledged to be responsive and empathetic. Although as one full-time clinical officer observed below, his job enabled and required empathy, making a comparison between full-time security officers and full-time health workers a fraught endeavour:

*I want to give you a scenario. There is an officer who is coming in the morning to pick someone to go for labour. Obviously someone will not feel good; if it’s cold season someone is telling you, you have to work, you feel bad. The officer is doing what he is supposed to be doing because they have some work to do. But here is me [a clinical officer] who’s coming on very tender, asking them: “How are you?”, Wanting to listen to what they are going through. And when they are sick the relationship becomes a little bit better. But that does not make me better than the other [prison officers], it’s the references of our businesses what we have to do. [Clinical Officer, Facility 3]*

Notwithstanding variation among individual officers and the conditions under which they operated, in all four facilities prisoners described the attitudes of some officers as an explicit barrier to care. These attitudes ranged from weak responsiveness or apathy to more active gate-keeping and power-plays as described in the quotes below:

*Sometimes they only take me when the illness has progressed and when I am suffering. I have no right to do anything. [Female, Facility 2]*

*For them to take us [to the clinic], you go to the [officer] and kneel down then ask her to take you. [Female, Facility 1]*

*You tell officers that you are unwell, and they tell you that you didn’t register. So if you didn’t register, then they won’t take you. [Female, Facility 3]*

Health workers seemed to confirm that officer attitudes could act as barriers to care:

*The [officers] even say they hate bringing the [prisoners] to the clinic. […] I can say for me that’s the thing that I don’t like. [Clinical Officer, Facility 4]*

However, while all the female officers interviewed acknowledged prisoners’ access to care was limited, this was, without exception attributed by officers themselves to administrative structures and/or resource shortages.

*The health facilities, here can’t meet their demands, so as a result, they are taken to the General Hospital. But taking them there is a problem because transport is a problem. [Officer, Facility 4]*

### Emergency care vs continuity of care

The compounding effect of the above-mentioned logistical and attitudinal barriers to accessing healthcare had serious ramifications for emergency access to care (such as at night) as well as for continuity of care for those with chronic conditions such as tuberculosis (TB) and HIV as outlined in Table 3.

**Table 4:** Interactions between structural & relational factors influencing health

1. ***Nutritional Status: Prison rations, visitation, and group membership*** Women’s nutritional status was influenced by the interaction between prison rations, structural and administrative factors influencing visitation, and relationships formed in prison. Inadequate prison rations were a critical health concern and their paucity resulted in heavy dependency by inmates on donations from friends and family. However, many women reported rarely receiving visitors, citing a range barriers including the limited female-holding capacity of local prisons, and frequent transfers to prisons geographically removed from their place of residence. Such factors made the costs of travel for friends and family prohibitive. In this context, the formation of inmate social and/or cooking groups took on great significance. Officer-appointed groups such as those in Facilities 1 and 2 encouraged resource-sharing among women prisoners of differing socio-economic backgrounds with some protective effects vis-à-vis nutritional status. Conversely, in facilities where groups were self-formed and more self-interested, some women were more exposed to the compounding effects of limited prison rations and lack of visitation.
2. ***Health service access: prison resourcing, administrative bias and inmate-officer relations*** Women’s access to health services was shaped by a combination of prison resourcing, administrative bias and inmate-officer relationships. Basic availability of health services for female prisoners was weak due to the absence of internal health services in any of the women’s facilities. In some sites, moreover, priority was given to male prisoners in accessing already limited transport to external health centres. Compounding these barriers, women reported varied and often ad hoc officer responsiveness to requests to access healthcare, with positive responses often based on inmates’ wealth or long-term relationship with the officer. Women prisoners with no visible physical symptoms of ill-health, as well as those looking after children, reported particular difficulties in persuading officers to commit resources to helping them access services.
3. ***Victimisation: Wealth, power and resource grabs.*** Longer-serving female prisoners and those appointed to senior inmate ranks tended to have closer relationships with prison officers, a situation enabled and cemented by behaviours including collusion on ‘resource grabs’ from Church donations. Such behaviour helped to ensure ranking inmates accumulated tradable items that could be used to ensure officer responsiveness to later requests for help, including requests for access to healthcare. Non-ranking inmates and those with no access to tradable goods were, by contrast, prone to vicitimisation by both officers and senior inmates. Women with chronic conditions and those looking after children were particularly vulnerable to verbal and physical belittlement since (as a result of ill-health or childcare duties) they were often unable to complete mandated chores or labour duties. An officer culture that accepted the use of psychological and occasionally physical abuse as a means of discipline and control meant less powerful inmates had little or no recourse.

Access to health care in the night was described as difficult (at best) in all four sites. Protocol required inmates to first inform their cell captain, who would bang on the door to alert a Duty Officer. The Duty Officer was then responsible for waking (generally by phone call) the Officer in Charge who had to be physically present to authorise any unlocking of a cell. In three of the four sites, inmates described, unprompted, having been present in a cell when a cell-mate died.

***P:****There was an inmate that fell ill at night. We called [the officer], but they didn’t come. They didn’t even take her to the clinic, so she died. We called them for a long time, but by the time they came she had already died.*

***I:****Were you there when that was happening?*

***P:****Yes I was there.*

***I:****And did you see that inmate die?*

***P:****Yes I did.*

***I:****So what did they say?*

***P:****Nothing, they just took the body to the hospital. [Female, Facility 3]*

*Nights are very bad times. Sometimes we call [the officers], but they are nowhere to seen. It is not always that they come when called. In response [to our request], they would say: “We need to call such-and-such a person first.” So sometimes the person they want to call is sleeping, so they don’t come in time. If they do [come], then it is by God’s grace. If they take long to come, then that inmate would even die in the cell. You even see this inmate dying. So those of us that watch this person [dying], our hearts break. In such instances, we don’t even sleep; we are full of worrying thoughts that if others are dying like this, we may also die the same way. [Female, Facility 2]*

#### Health-seeking behaviours

Although many respondents articulated a clear desire to remain healthy, the constant barriers to healthcare care undermined some women’s interest in trying to look after their health:

*Here in prison, [health] is not important because I am suppressed in everything; the sleeping arrangement, the heavy chores we do, everything. I don’t have the strength to manage my TB well. If we were taken care of, then I would have loved to improve my health. If they cared about taking us to the hospital, because they act as our parents. But we tell them about how we feel and they don’t take us to the hospital. So nothing is happening. [Female, Facility 2]*

*If one falls ill at night, like I fell very sick last night, I just suppressed the feeling, because I thought of shouting for help from the officer. I just sat back. Prison life is tough. [Female, Facility 2]*

#### Service quality and responsiveness

With many women more focused on concerns relating to health service access fewer women described differences in the quality and responsiveness of those services. Where women were able to access external (MOH) facilities, several respondents described healthcare workers advocating for them to receive better or more routine access to care.

*The treatment is good here. It is the same as before I was incarcerated. [Female, Facility 3]*

*The medical personnel actually tell the prison officers if they have not followed the appointment date. Sometimes we skip the appointment dates. The officers say there is no manpower. So when we go to the hospital after due date, the medical personnel now tell the prison officers off. [Female, Facility 2]*

However, issues relating to lack of confidentiality and privacy, particularly in relation to security protocol that required officers to sit in on consultations were noted by several respondents:

*P: I received better treatment when I was outside, because outside there’s freedom and you speak for yourself.*

*I: Here you don’t speak for yourselves?*

*P: We do but with fear because the bwana [boss/officer] always sits next [to me]. [Female, Facility 3]*

Drug stock outs, limited equipment and a lack of health personnel (or at least limited opening hours) at the adjacent primary health clinics were also frequently mentioned problems. Significantly, several inmates and officers commented that many referrals to hospital services made by these clinics (and all the attendant problems in sourcing transport) could be avoided if the clinics themselves were better staffed and equipped.

### Looking after children

Interviews surfaced a high proportion of women struggling to look after young children in prison. Seven of our 23 inmate respondents (30 %) had a child living with them at the time of study. Childrens’ ages ranged from 3 months to 4 years old. These inmates consistently described their children’s acute vulnerabilities in relation to the same nutritional, social and health service issues described above, summarised by one respondent who commented:

*I have a child who is 3 years old. We are suffering here together. [Female, Facility 4]*

#### Children's nutrition

Finding sufficient and appropriate food and clean clothes for infants was described as a daily struggle. All women noted the inappropriate types and quality of food for infants and described the difficulty of maintaining breastfeeding when their own nutritional status was so weak; finding supplemental milk when they were unable to breastfeed appeared to be a matter of chance.

*We just eat daga [porridge], kapenta and beans without cooking oil. And with children it’s difficult to feed a child with such. [Female, Facility 3]*

*On the children’s side, mothers can’t afford to feed children the required food. So children can be sick of malnutrition. We need a lot of help when it comes to the babies’ food. We are given the same foods [as the adults] and babies can’t take it, and as a result they tend to have illnesses. [Female, Facility 3]*

None of the women with children reported receiving regular visitors to assist with their childrens’ needs, although several described having (unsuccessfully) requested transfers to prisons nearer their family in order to access their support. In Facility 1, two women reported the ‘buffering’ effect of belonging to mixed group since group members would sometimes share food. In Facilities 2 and 4, however, respondents described the impossibility of asking other women for food without something to exchange. In all four sites, women with children reported Church and NGO donations were fundamental to fulfilling their children’s basic needs, although even these were often insufficient.

*Sometimes people from church come to help and we share things out. But for those like me with a baby - this baby was born here, he is three months old – when the Church bring soap you find that there will be a shortage, because we need to wash baby nappies. [Female, Facility 1]*

#### Developmental concerns

The poor developmental environment – particularly lack of physical freedom for young children was a common concern.

*There is no space, like for my child, she likes moving up and down. But there is no space for her to move up and down, so she would feel bad, wanting to move about here and there. Then you start quarrelling with others inside [Female, Facility 2]*

Women also drew attention to the impact on children of watching ‘bad aspects’ of prison life or being verbally or physically abused by other inmates.

*I have a child. Sometimes she can do something that will upset an inmate. Then they will talk to her in a rude manner and sometimes even hit her, saying I don’t teach her manners. [Female, Facility 1]*

*When a new inmate comes into prison they are lectured by officers and captains in presence of the children. They are made to dance like they are at the initiation ceremony while children are watching. Children are always there watching and they start copying, the dancing and all the words that are used, children will be there watching. [Female, Facility 3]*

#### Pregnant women and childrens’ access to healthcare

All seven women with children reported challenges in accessing timely health services for their children.

*Mothers and pregnant women are not supposed to be here. It is very difficult for them [officers] to take us to the clinic, especially just for the children and women in labour. [Female, Facility 1]*

*My daughter has a cough so there’s a time when it became worse. I told the [officers], but they said we wait and see how she will be by tomorrow. Then in the night she became worse. We started calling for the officer, when they came they were upset saying the officer-in-charge gets upset when they open the cells. Once we are locked she doesn’t allow that. [Female, Facility 3]*

One respondent who had previously had TB additionally described particular difficulty looking after her child while she was herself ill. In addition to the burdens of sourcing food she noted intense stigma from both officers and fellow prisoners in relation to her TB status.

*Inmates also used to treat me like the officers, saying l would infect them if they came near me, and also never allowed me to touch their babies because l would infect them. I was also put in the isolation room, with my baby who wasn’t sick at that time. Female, Facility 1]*

## Discussion

This study is the first to systematically examine interactions between structural, social and relational factors influencing female prisoners’ health and access to health care in Zambia. It is only the second independent study of female prisoners in Zambia following a seminal Human Rights Watch report [31] and Todrys and Amon’s [32] subsequent paper which highlighted the dire conditions of Zambian prisons. Our findings confirm disturbing features of women prisoners’ experience previously outlined in those works such as the health risks inherent in the poor physical condition of cells including poor or non-existent ventilation and sub-optimally maintained sanitation facilities; the inadequacy and insufficiency of prison rations for meeting womens’ – and their childrens’ – nutritional needs; and problematic access to healthcare. Such findings suggest that in these domains, at least, there has been little material progress since 2010.

The unique inclusion of both female prisoners and non-ranking facility-level officers in our interview schedule enabled triangulation of perspectives that provided insights into how interactions among prison management approaches, prisoner relationships, and social inequalities are leading to deteriorations in female prisoners’ health and healthcare access. Key amongst these were the intersecting role of: i) basic resourcing - inadequacies of the prison system, ii) structural (albeit) unintentional prioritisation of the health needs of male prisoners, and iii) the chronic and unchecked pattern of both inmate-led and officer-led victimisation.

A series of specific interactions based on data presented here are detailed in Table 4 and encompass the intersection between poor prison rations, impediments to family visitation and womens’ membership in (officer- or self-appointed) cooking groups; the interaction between lack of in-house health services, privileged male prisoner access to transport, and a general lack of responsiveness to health issues among female prison officers; and the interaction between female inmates’ comparative wealth on entry to prison, the appointment of senior ‘special stage’ inmates, and an officer culture tolerant of victimisation.

Our findings shine a particularly harsh light on the impact of interactions between structural and relational features of prison on the health and access to healthcare of female prisoners living with children, as well as those separated from their children. Pregnant women and those with newborns have specific health needs and findings from this study demonstrated that the hygiene, gynaecological and reproductive needs of women prisoners were frequently overlooked. Although not a specific focus of this study, our findings also highlight the potentially damaging effects of inappropriate nutrition and the stressful prison environment on the physical and mental development of children growing up in prison [33].

The trauma of social isolation experienced by women separated long-term from their families and children was also noted. Research from other settings suggests that the prohibition of, or limitation (deliberate or coincidental) of visitation rights is more psychologically damaging to mothers than to unattached, single prisoners [34, 35]. Our data suggest that in the Zambian prison setting, mothers from socio-economically marginalised backgrounds were, in addition to experiencing isolation from family and friends, more vulnerable to chronic victimisation by senior inmates and officers due to their inability to always comply with prison schedules or labour duties while looking after their childrens’ needs.

The small overall number of women prisoners in Zambia (and regionally) means that gender specific concerns and gender mainstreaming in prison policy or officer training has not been a priority for ZPS to date. With the exception of provision for female prison officers, structural conditions, security procedures, healthcare services, visitation guidelines and daily activities remain derived from a largely male model. Despite this, women prisoners are clearly afforded fewer service provisions and less programming that those assigned to their male counterparts. These factors, as well as the interactions outlined above all point to the way in which Zambian prisons are exacerbating the vulnerabilities and inequities experienced by female inmates.

### Strengths and limitations

We adopted a methodologically rigorous approach based on representative site selection, representative sampling of prison officers, and random sampling of adult female prisoners. Collection of qualitative data from both inmates and officers helped us test the claims of various respondents and enhanced the validity of our findings. Unusually in the context of prison research in this region, we obtained permission to record and transcribe all interviews, enabling in-depth analysis based on verbatim (anonymised) transcripts. With more time and resources, a larger sample of prison sites and informants including open-air prisons and juvenile inmates would have strengthened the study’s representativeness. We note that there is only one study including consideration of juvenile female inmates in Zambia to date [36]. Despite these limitations, the sample was deemed appropriate given the study’s stated aims and in the context of broader security and logistical constraints of the prison setting. Clearly the findings from this study carry most relevance to the Zambian prison setting, although some do highlight issues likely to be of concern across the region more broadly.

## Conclusion

This systems-oriented analysis revealed a series of dramatic health needs among Zambia’s female prisoners, including lack of access to basic sanitation and limited access to routine health services including for antenatal and reproductive health, TB and HIV. The study revealed that Zambian female prisoners’ health and access to healthcare is influenced by interactions between generally weak resourcing for prisoner health, administrative biases that prioritise male access, and a prevailing organisational and inmate culture, with the dynamic relationship between these both creating and exacerbating general and gender-specific health risks. These findings highlight the urgent need for investment in structural improvements in health service availability for female prisoners but also interventions to reform the organisational culture which shapes officers’ understanding and responsiveness to female prisoners’ health needs.

## Abbreviations

CAPAH: Coalition of Parliamentarians Against HIV/AIDS
HIV: Human immune deficiency virus
MCDMCH: Ministry of Community Development Mother and Child Health
MHA: Ministry of Home Affairs
MOH: Ministry of Health
TB: Tuberculosis
UNODC: United Nations Office on Drugs and Crime
ZPS: Zambia Prisons Service
